# Unilateral Vocal Cord Paralysis in a Patient With Acute Viral Hepatitis

**DOI:** 10.7759/cureus.13399

**Published:** 2021-02-17

**Authors:** Ayesha Malik, Mohammad Abdullah, Manahil Chaudhry, Amna Murtaza, Ayesha Kanwal

**Affiliations:** 1 Medicine, Hameed Latif Hospital, Lahore, PAK; 2 Medicine, CMH Lahore Medical College and Institute of Dentistry, Lahore, PAK; 3 Otolaryngology, CMH Lahore Medical College and Institute of Dentistry, Lahore, PAK

**Keywords:** vocal cord paralysis, viral hepatitis a, direct laryngoscopy, infectious hepatitis

## Abstract

Hepatitis A virus is a leading cause of acute infectious hepatitis worldwide. The infection is characterized by a self-limiting course, and rarely has there been any occurrence of chronic sequelae or extra-hepatic manifestations. We report a case of unilateral vocal cord paralysis in a patient with acute hepatitis A.

## Introduction

Hepatitis A virus (HAV) is an RNA virus that causes acute infectious hepatitis. It has a worldwide annual prevalence of 1.5 million cases [1]. The virus causes liver injury by an immune-mediated mechanism [2]. It majorly presents as a self-limiting disease and is characterized by low-grade fever, nausea, vomiting, and diarrhea [3]. Unlike hepatitis B and hepatitis C virus (HCV), there are seldom any reports of chronic sequelae originating from an acute HAV infection [4,5]. The reporting of any extra-hepatic complication with HAV is even rarer. Here, we report the case of a 25-year-old male who presented with unilateral vocal cord paralysis (UVCP) during acute viral hepatitis A.

## Case presentation

A previously healthy, 25-year-old male presented to our outpatient department with a four-day history of intermittent high-grade fever followed by a two-day history of vomiting, yellowing of sclera, decreased oral intake, malaise, and hoarseness of voice. There was no history of cough, flu-like symptoms, neck pain, or rash. On examination, scleral icterus, mild right hypochondrium tenderness on deep palpation, and hepatomegaly were noted. The liver had smooth margins and was firm in consistency. No splenomegaly or lymphadenopathy was appreciated. Neurological examination was unremarkable with no altered mentation or neurological deficits. Cranial nerve examination was normal. He had normal tongue movements and the uvula was central. The sensory and motor functions of limbs were intact.

He was admitted for evaluation and his baseline laboratory investigations were ordered. He was started on supportive treatment with intravenous fluids and anti-emetics. His blood work revealed alanine aminotransferase 1,650 U/L (normal range: 0-42 U/L), alkaline phosphate 1,085 U/L (normal range: 65-300 U/L), and serum total bilirubin 191 µmol/L (normal range: 0-17 µmol/L). His blood count, coagulation profile, and renal function test were within normal limits. Serology for hepatitis B surface antigen and antibodies for hepatitis C were negative. Abdominal ultrasound revealed hepatomegaly. Serology for hepatitis A revealed significant titers for IgM depicting an acute hepatitis A infection.

Pertaining to his hoarseness, it was initially attributed to persistent vomiting, but when it continued even after his vomiting was settled, he was referred to the otolaryngology team who planned a fiberoptic direct laryngoscopy (FODL). Examination via FODL revealed left vocal cord paralysis (Video 1). No other abnormality or growth was detected. A further probe into the cause of this lesion was carried out. This was the first presentation with hoarseness and he had never had such symptoms in the past. A contrast-enhanced computed tomography of the neck and chest was carried out which revealed no lesions.

**Video 1:** Unilateral (left) vocal cord paralysis. The video shows left vocal cord paralysis. During the procedure, we asked the patient to verbalize the letter “E” to look for vocal cord movement. It can be seen that the left vocal cord (on the viewer’s left side) is immobile. The volume has been muted due to background noise.

He clinically improved and his serial liver function tests showed improvement. He was discharged on symptomatic treatment. He was called for a follow-up six weeks later. Upon follow-up, his liver function tests had almost normalized but his hoarseness persisted. He was called for a second follow-up at three months and offered a magnetic resonance imaging (MRI) of the brain to look for any pathology. The MRI study was also normal. He was then advised a further follow-up at six months.

## Discussion

We present a case of UVCP (left) in a setting of acute hepatitis A infection. The vocal cords are supplied by the recurrent laryngeal nerve, a branch of the vagus nerve. Vocal cord paralysis is characterized by loss of vocal cord adduction and abduction and can be secondary to mechanical fixation or neuropathy involving recurrent laryngeal or vagus nerve [6]. UVCP causes problems related to phonation, deglutition, and respiration. Patients present with characteristic hoarseness of voice, voice fatigue, cough, aspiration, and swallowing difficulties [7]. Among viruses, Epstein-Barr virus, varicella-zoster virus, and herpes simplex virus infection have been linked to the development of vocal cord paralysis [8-10]. Although the pathogenesis behind this condition is not fully known, two mechanisms have been proposed. First, direct inflammation and infection of the nerves by the virus, and second, induction of non-specific inflammatory response secondarily involving a nerve [11].

There is no established association of HAV with vocal cord paralysis; however, there has been reports of mononeuritis (involving lateral cutaneous nerve of the thigh, median nerve, axillary nerve, and facial nerve) associated with acute HAV infection [12]. In addition, Prasad and colleagues [13] described a case of an eight-year-old boy who developed palatal palsy in a setting of an acute HAV infection. To our knowledge, the only likely correlation of UVCP with HAV in previous literature was the mention of George London, an opera singer, who developed UVCP likely due to HAV [11]. Hence, the presentation is a clinical rarity.

## Conclusions

Our case report presents an extremely rare incidence of UVCP secondary to acute hepatitis A infection. Post-viral recurrent laryngeal nerve involvement should be considered after all possible causes of vocal cord paralysis have been ruled out.
